# Stability selection machine learning identifies biomechanical markers for subclinical idiopathic corneal endothelial abnormalities

**DOI:** 10.3389/fmed.2026.1899851

**Published:** 2026-07-28

**Authors:** Chengjie Feng, Miaomiao Chi, Shaofeng Gu, Jing Hong

**Affiliations:** Department of Ophthalmology, Peking University Third Hospital, Beijing, China

**Keywords:** CCT-stratified analysis, corneal biomechanics, Corvis biomechanical index, endothelial cell density, noninvasive biomarkers

## Abstract

**Background:**

The early detection of idiopathic corneal endothelial compromise, a common yet underdiagnosed pre-cataract finding, remains a challenge. This study aimed to investigate the association between corneal biomechanical parameters and endothelial status, identify potential biomechanical markers, and explore the biomechanical manifestations of idiopathic endothelial impairment.

**Methods:**

In this cross-sectional study, cataract patients with normal endothelium or idiopathic endothelial abnormality underwent Corneal Visualization Scheimpflug Technology (Corvis ST) tonometry and specular microscopy. Beyond conventional statistics, we employed stability selection—a machine learning method with explicit error control—to identify the most reproducible predictors from a multitude of biomechanical parameters, while rigorously accounting for central corneal thickness (CCT) via stratified analysis and propensity score matching (PSM).

**Results:**

Among 241 patients (35 with endothelial abnormality), five biomechanical parameters (SSI, HCR, A2L, A1T, cTBI) significantly differed between groups, even after PSM for CCT. Stratified analysis revealed the strongest biomechanical-endothelial correlations specifically within the mid-range CCT (530–570 μm). A2L was identified as an independent protective factor (OR: 0.202, 95%CI: 0.051–0.731, *p* = 0.018). Stability selection confirmed A2L and cTBI as the most robust markers. A novel DASC model (incorporating A2L, DA, SSI, and CCT) achieved an AUC of 0.711 for detecting endothelial abnormalities, comparable to the cTBI score (AUC: 0.683, 95%CI: 0.565–0.800).

**Conclusion:**

Corneal biomechanics reflect early endothelial alterations. A2L, cTBI, and the DASC model serve as promising, noninvasive tools for risk stratification. Our findings suggest that subclinical endothelial impairment manifests as reduced corneal stiffness-related response and impaired deformation behavior.

## Introduction

The corneal endothelium is a non-regenerative monolayer essential for maintaining corneal transparency through its pump function (1–6). Endothelial cell density (ECD) naturally declines with age, yet most individuals retain ECD > 2000 cells/mm^2^ throughout life (7). Clinical decompensation usually occurs only when ECD falls to 400–700 cells/mm^2^ (8–10), but patients with intermediate counts between age-related lower limits and conventional “compensatory” cut-offs may already exhibit functional impairment and reduced surgical tolerance, even in the absence of overt edema (11–13).

A subset of such “subclinical” endothelial impairment is idiopathic: patients have no history of glaucoma, intraocular surgery or corneal dystrophy and are identified only during routine pre-cataract assessment, making their surgical vulnerability difficult to anticipate. In daily practice, endothelial health is assessed mainly by ECD, coefficient of variation (CV) and hexagonality (HEX) on specular microscopy (14–16), sometimes complemented by pachymetry or anterior segment OCT. These morphological indices, however, provide limited insight into functional integrity (17), and may miss early or borderline dysfunction in asymptomatic eyes. There is thus a clear need for a non-invasive, functionally oriented assessment that can flag high-risk corneas before clinical decompensation.

At the mechanistic level, our recent experimental work (18) has shown that extracellular matrix (ECM) stiffness directly governs corneal endothelial barrier integrity through YAP-mediated stabilization of the tight-junction protein ZO-1: softer ECM disrupts ZO-1 localization and increases permeability even before frank cell loss or morphological change. This finding provides a biologically plausible basis for expecting that alterations in corneal stiffness may mirror early endothelial vulnerability.

Clinically, the Corneal Visualization Scheimpflug Technology (Corvis ST; OCULUS Optikgeräte GmbH, Wetzlar, Germany) enables *in vivo*, dynamic and global assessment of corneal biomechanics (19–21), and its parameters and composite indices have been validated in ectasia screening (22, 23). Advanced endothelial failure is known to induce stromal edema and alter deformation behavior (22, 24), and subclinical stromal changes have been detected biomechanically in Fuchs endothelial corneal dystrophy (FECD) (25). However, most prior studies have focused on genetically or clinically defined dystrophies; little is known about the biomechanical signature of unexplained, asymptomatic endothelial impairment, and the diagnostic or predictive potential of Corvis ST metrics in this high-risk but understudied population remains unclear.

A further challenge is methodological: correlating numerous, correlated biomechanical variables with clinical outcomes in modest samples carries a high risk of false-positive findings and poor reproducibility. Conventional regression or single-run LASSO may overfit and inflate spurious associations. To address these gaps, we combined central corneal thickness (CCT)–stratified analysis with stability-selection–based machine learning, an error-controlled approach that estimates each variable’s selection probability and bounds the expected number of false discoveries. We hypothesised that this framework would allow us to identify which Corvis ST parameters show the strongest and most reproducible association with idiopathic subclinical endothelial impairment, thereby providing credible non-invasive biomarkers to support early risk assessment and surgical planning.

## Methods

### Study population

This single-center cross-sectional study included patients with normal ECD as well as those with reduced ECD of unknown etiology.

### Inclusion criteria

The study enrolled patients diagnosed with age-related cataract according to the *AAO Preferred Practice Pattern® for Cataract in the Adult Eye* (26), including features such as age over 50 years, progressive lens opacity on slit-lamp examination, and corresponding visual impairment not attributable to other ocular pathology. All subjects completed standardized assessments, including slit-lamp biomicroscopy, Corvis ST measurements (three successful scans), and specular microscopy (≥100 analyzable cells).

### Exclusion criteria

Patients with ocular conditions known to affect corneal biomechanics or endothelial morphology were strictly excluded from the study. Exclusion criteria included: (1) a confirmed diagnosis of specific corneal diseases (e.g., keratitis, keratoconus, corneal endothelial dystrophies, or any other clearly diagnosed corneal pathology) or slit-lamp biomicroscopy revealing stromal edema, corneal opacity, infiltrates, scars, or other abnormal findings suggestive of structural compromise; (2) diagnosed glaucoma (either open- or closed-angle) or any condition associated with sustained elevation of intraocular pressure; (3) any form of intraocular inflammatory disease that may cause endothelial damage; (4) a history of ocular surgery involving the cornea or limbus, regardless of whether intraocular entry was involved; (5) contact lens wear—either soft or rigid—within 2 weeks prior to Corvis ST measurement; (6) inability to cooperate with corneal biomechanics or endothelial imaging assessments.

For patients with both eyes meeting the inclusion criteria, one eye was randomly selected for analysis to ensure data independence. Eyes with ECD < 2000 cells/mm^2^ and meeting the above criteria were classified as idiopathic endothelial abnormality (11, 27, 28).

This study was approved by ethics committee of Peking University third Hospital (IRB No.: M2022834). All methods were performed in accordance with the Declaration of Helsinki.

### Clinical examination

All participants underwent standardized evaluation including refraction, slit-lamp biomicroscopy, non-contact tonometry, and specular microscopy (ECD, CV, HEX). Central corneal thickness (CCT) was measured with ultrasound pachymetry. Measurements were performed by trained technicians following manufacturer protocols.

All participants underwent a standardized ophthalmic examination, including manifest refraction, slit-lamp biomicroscopy, non-contact tonometry, and corneal endothelial assessment by specular microscopy. Slit-lamp examination was performed to evaluate corneal transparency, anterior segment abnormalities, and the presence of clinically evident corneal edema or endothelial disorders. Specular microscopy was used to obtain endothelial cell density (ECD), coefficient of variation in cell area (CV), and percentage of hexagonal cells (HEX). Central corneal thickness (CCT) was measured using ultrasound pachymetry. All measurements were performed by trained technicians according to the manufacturers’ protocols, and only examinations with acceptable image quality were included in the analysis.

### Corneal biomechanics

Corneal biomechanical data were obtained using Corneal Visualization Scheimpflug Technology (Corvis ST; OCULUS Optikgeräte GmbH, Wetzlar, Germany), which records the dynamic corneal response to a standardized air puff using ultra-high-speed Scheimpflug imaging. Corvis ST-derived parameters were interpreted as dynamic corneal response or deformation response indices. Tomographic data and composite indices were obtained using Pentacam. Parameters derived from established biomechanical models (cCBI, cTBI and BAD. D) were classified as composite indices, others were classified as “raw features.” Only examinations meeting quality-control criteria, including acceptable Pentacam quality specification and Corvis waveform score A, were included.

### Statistical analysis

All statistical analyses were conducted using R software (version 4.5.1).

#### Description analysis

Continuous variables were tested for normality using Shapiro–Wilk normality test and compared using t-tests or Wilcoxon rank-sum tests as appropriate, while categorical variables were assessed with chi-square tests.

#### Matching analysis

Propensity score matching (PSM) was used for sensitivity analysis to control for CCT. To evaluate the quality of matching, standardized mean differences (SMDs) and empirical cumulative distribution functions (eCDFs) were calculated. A post-matching SMD approximating 0.2 was considered indicative of adequate balance.

#### Continuous CCT interaction analysis

Separate logistic regression models were applied for each key biomechanical parameter, including the parameter-by-CCT interaction term and adjusting for age and bIOP. All continuous variables were standardized before model fitting, and ORs for interaction were reported per 1-SD increase.

#### Correlation analysis

The cut-offs were selected to separate relatively thin, mid-range, and relatively thick corneas around the commonly observed CCT range while maintaining a reasonable sample size in each stratum. Spearman correlation was applied. Correlation strength was interpreted as follows: |*ρ*| < 0.2, very weak; 0.2 ≤ |*ρ*| < 0.4, weak; 0.4 ≤ |*ρ*| < 0.6, moderate; and |*ρ*| ≥ 0.6, strong (29).

#### Regression and modeling

Multivariable logistic regression was performed with events-per-variable ≈10. Candidate predictors included clinically relevant variables and stability-selected features. Collinearity was checked using variance inflation factors (VIF < 2). Model performance was assessed using AUC with bootstrap resampling.

#### Machine learning

To improve reproducibility, we used stability selection (50% subsampling, B = 1,000, LASSO base learner), designating variables with selection probability ≥0.80 under PFER ≤1 as stable predictors. Raw features and composite indices were analyzed in separate runs.

## Results

### Baseline characteristics of the elderly population

**Figure 1:**
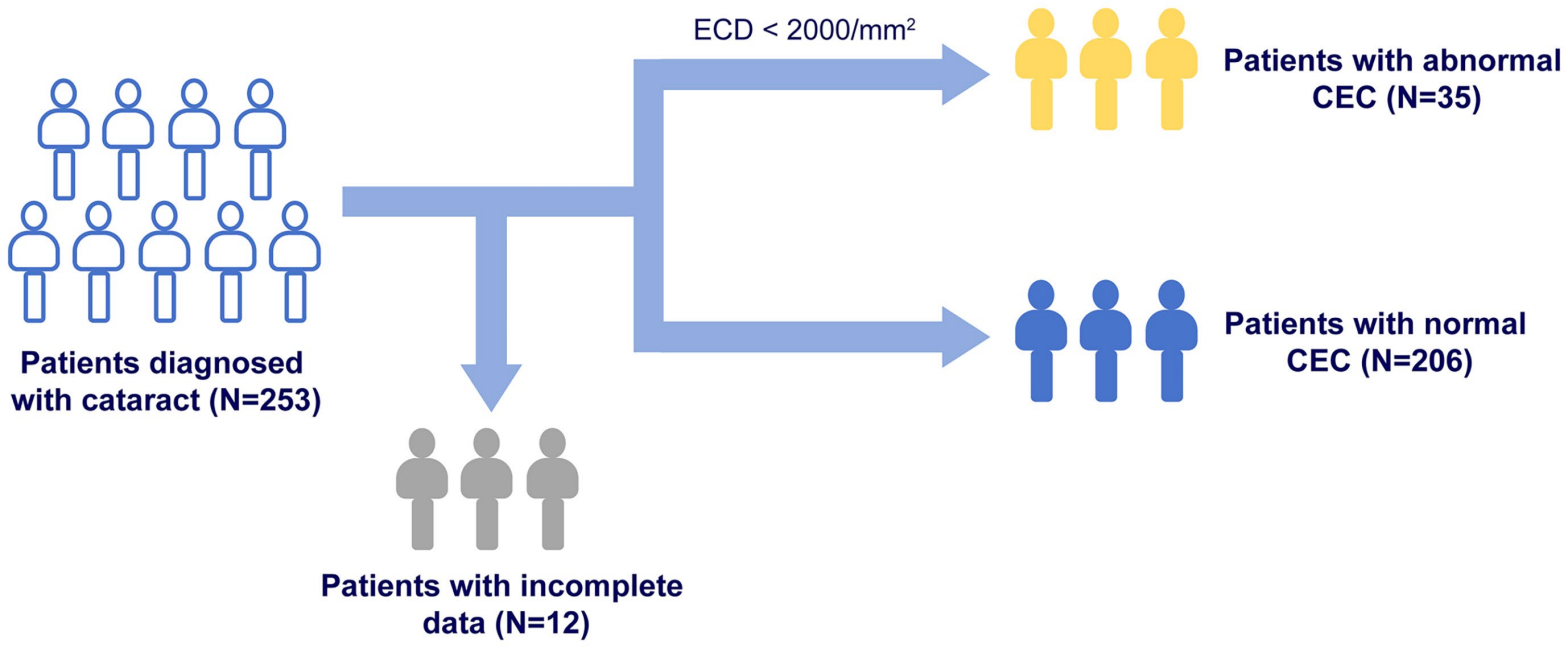
Participant selection and grouping flowchart. A total of 253 patients diagnosed with cataract were initially included. After excluding 12 patients due to incomplete data, 241 patients were classified into two groups according to corneal endothelial cell (CEC) status. Patients with endothelial cell density (ECD) < 2000 cells/mm^2^ were categorized as the *abnormal CEC group* (*N* = 35), and the rest were classified as the *normal CEC group* (*N* = 206).

A total of 253 patients diagnosed with cataract between July 1, 2024 and July 1, 2025 were included in this cross-sectional study. Two hundred forty-one patients with complete clinical records were finally enrolled in this study (Figure 1), of whom 35 were identified as having abnormal corneal endothelium. As shown in Table 1, patients with endothelial dysfunction exhibited significantly thinner CCT. As expected, CEC abnormal group exhibited impaired ECD, while HEX and CV showed no significant group differences.

**Table 1:** Baseline characteristics of the elderly population.

Variables	Healthy CEC	Abnormal CEC	p.overall
	*N* = 206	*N* = 35	
Sex (Male)	92 (44.66%)	10 (28.57%)	0.110
Age	69.00 (61.00, 75.00)	70.00 (57.00, 78.00)	0.803
Category	101 (49.03%)	18 (51.43%)	0.937
AL	23.63 (22.90, 24.75)	23.92 (23.30, 25.21)	0.058
CCT	556.49 ± 33.17	539.00 (520.50, 570.50)	0.011
bIOP	14.04 ± 2.15	13.56 ± 2.59	0.313
nctIOP	15.64 ± 2.55	15.00 (13.00, 16.50)	0.156
ECD	2,765 (2,602, 2,927)	1514.00 (830.50, 1805.50)	<0.001
HEX	67.00 (63.00, 70.00)	65.00 (61.00, 69.00)	0.285
CV	29.00 (27.00, 33.00)	29.00 (24.00, 37.00)	0.362

Comparison of clinical baseline characteristics and endothelial parameters between eyes with normal and abnormal corneal endothelium. Data are presented as n (%) for categorical variables and as mean ± SD or median (IQR) for continuous variables, depending on distribution. Abnormal CEC was defined as ECD < 2000 cells/mm^2^.

AL, Axial length; CCT, central corneal thickness; bIOP, Biomechanically corrected intraocular pressure; nctIOP, Non-contact tonometer intraocular pressure; ECD: endothelial cell density; HEX: hexagonality; CV: coefficient of variation in cell size.

### Eyes with endothelial abnormalities exhibit altered corneal biomechanical profiles

Five corneal biomechanical parameters showed statistically significant differences between patients with normal and abnormal CEC. Patients in the low ECD group demonstrated significantly lower values of Stiffness Parameter at First Applanation (SSI, *p* = 0.028), Highest Concavity Radius (HCR, *p* = 0.010), A2 Length (A2L, p = 0.010), and A1 Time (A1T, *p* = 0.020), as well as higher values of corrected tomographic biomechanical index (cTBI, *p* = 0.001). (Supplementary Figure S1).

A sensitivity analysis using propensity score matching (PSM) was performed to isolate the confounding influence of CCT. After 1:5 matching, a total of 210 eyes (35 cases and 175 matched controls) were included for further analysis. PSM based on CCT achieved moderate covariate balance, with the SMD of CCT improving from −0.4934 before matching to −0.2154 after matching. The baseline characteristics (especially CCT) of the matched groups revealed no significant differences (Table 2). The five previously identified biomechanical parameters (SSI, HCR, A2L, A1T, and cTBI) remained significantly different between the two groups (Table 2). These results reinforce the robustness of five biomechanical markers in differentiating corneas with abnormal endothelial conditions even after adjustment for structural differences.

**Table 2:** Comparative analysis of baseline characteristics and biomechanical profiles after matching.

Variables	Cataract	Abnormal CEC	p.overall
	*N* = 175	*N* = 35	
Sex (Male)	81 (46.29%)	10 (28.57%)	0.081
Age	70.00 (61.00, 76.00)	70.00 (57.00, 78.00)	0.691
AL	23.57 (22.83, 24.76)	23.92 (23.30, 25.21)	0.390
CCT	552.00 (530.00, 570.00)	539.00 (520.50, 570.50)	0.207
ECD	2762.00 (2596.50, 2911.00)	1514.00 (830.50, 1805.50)	<0.001
HEX	67.00 (63.00, 70.00)	65.00 (61.00, 69.00)	0.276
CV	30.00 (27.00, 33.00)	29.00 (24.00, 37.00)	0.365
bIOP	14.12 ± 2.20	13.56 ± 2.59	0.244
nctIOP	15.50 (13.50, 17.00)	15.00 (13.00, 16.50)	0.401
SSI	1.10 (0.93, 1.23)	0.98 (0.81, 1.19)	0.020
cTBI	0.10 (0.03, 0.22)	0.22 (0.09, 0.81)	0.003
HCR	6.93 (6.57, 7.33)	6.65 (6.26, 7.16)	0.020
A2L	1.99 (1.75, 2.09)	1.76 (1.60, 2.09)	0.023
A1T	7.31 (6.52, 8.16)	6.76 (5.76, 7.92)	0.040

After 1:5 propensity score matching based on CCT, 175 eyes with healthy endothelium and 35 with abnormal endothelium were analyzed. Baseline characteristics and statistically significant biomechanical parameters are shown. After matching, baseline characteristics (especially CCT) of the matched groups revealed no significant differences. Data are expressed as mean ± SD or median (IQR) depending on distribution.

AL, Axial length; CT, central corneal thickness; ECD, endothelial cell density; HEX, hexagonality; CV, coefficient of variation in cell size; bIOP, Biomechanically corrected intraocular pressure; nctIOP, Non-contact tonometer intraocular pressure; SSI, Stiffness parameter at first applanation; cTBI, corrected tomographic biomechanical index; HCR, Radius of curvature at highest concavity; PD, Peak distance between two corneal bending points at highest concavity; A2L, Length of flattened cornea at second applanation; A1T, Time from start until first applanation.

Continuous CCT interaction analyses were performed to assess whether CCT modified the association between biomechanical parameters and abnormal endothelial status. After adjustment for age and bIOP, no significant CCT-by-parameter interactions were observed for A2L, SSI, HCR, A1L, or cTBI (all P for interaction > 0.05; Supplementary Table 1). These findings suggest that the observed biomechanical–endothelial associations were not significantly modified by CCT on the multiplicative scale.

### Biomechanical–endothelial correlations are stratified by corneal thickness

**Figure 2:**
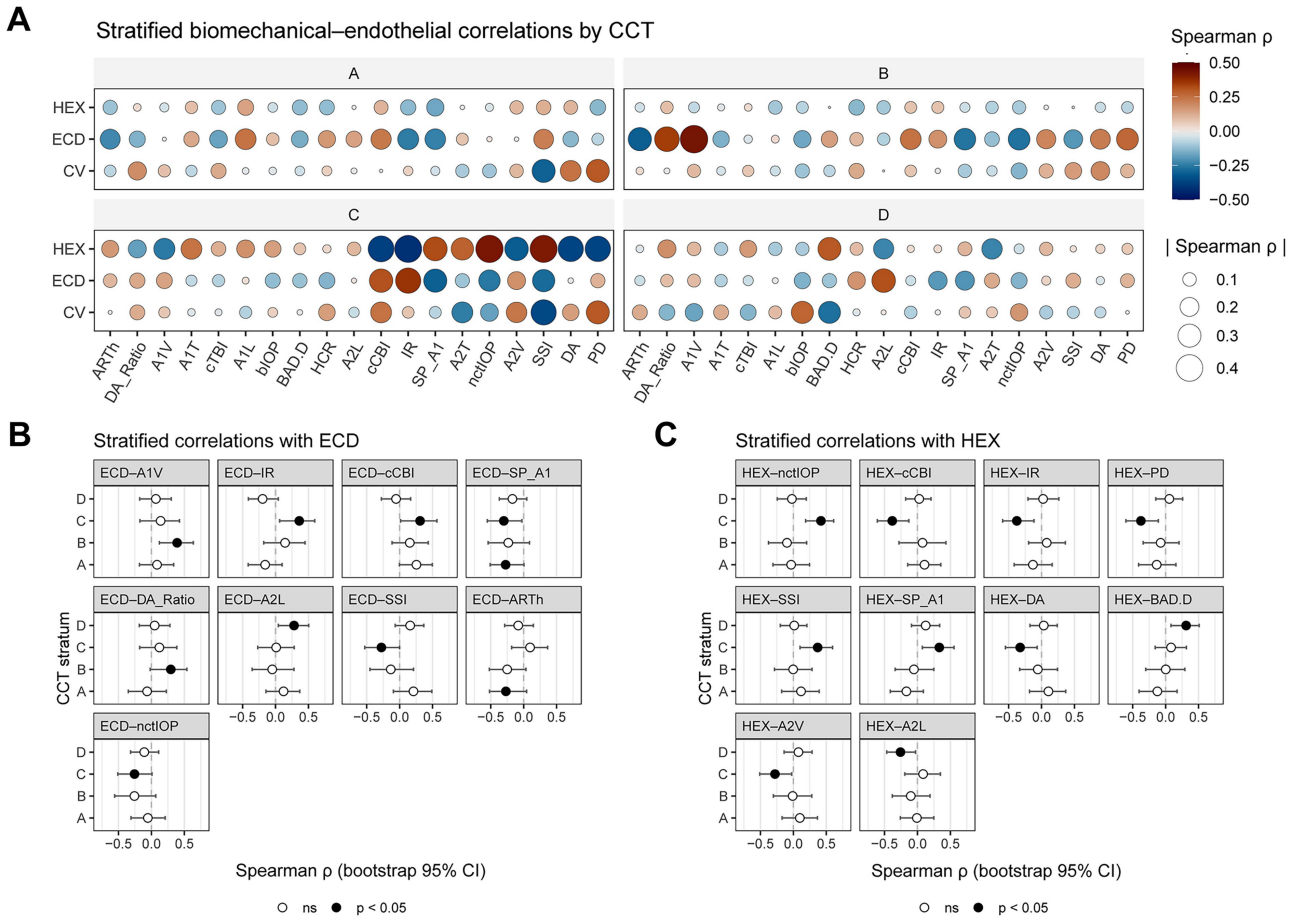
Stratified biomechanical–endothelial correlation analysis by central corneal thickness (CCT). **(A)** Heatmap of stratified correlations. Spearman correlation coefficients (*ρ*) between endothelial parameters [ECD, HEX, CV] and biomechanical parameters are shown across four CCT strata (A–D). Color represents correlation direction and strength, while circle size represents absolute *ρ*. **(B)** Stratified correlations with ECD. Forest plots of selected ECD correlations with biomechanical parameters across CCT strata. Points represent correlation coefficients; error bars indicate bootstrap 95% confidence intervals. Filled circles indicate significant correlations (*p* < 0.05). **(C)** Stratified correlations with HEX. Forest plots of selected HEX correlations with biomechanical parameters across CCT strata. Plotting conventions are the same as in **B**. CCT strata were defined as A (<530 μm), B (530–550 μm), C (550–570 μm), and D (>570 μm). ECD, endothelial cell density; HEX, endothelial cell hexagonality; CV, coefficient of variation.

Significant associations clustered in the mid-range CCT strata—Groups B (530–550 μm) and C (550–570 μm)—while patterns were sparse in the thinnest (A) and thickest (D) corneas (Figure 2A).

In Group B (CCT: 530–550 μm), ECD correlated positively with A1V and DA Ratio, indicating that higher endothelial cell density accompanies faster first-applanation velocity/greater central deformation pattern in this CCT range, which correspond to better central deformation response.

In Group C (CCT: 550–570 μm), HEX was positively correlated with SSI (*ρ* = 0.419) and nctIOP (*ρ* = 0.426), and weakly negatively correlated with IR (*ρ* = −0.419), cCBI (*ρ* = −0.383), and PD (*ρ* = −0.368). This pattern reflects a biomechanical profile of greater stiffness-related response and compact deformation in eyes with better endothelial morphology.

In Group D (CCT: 570–645 μm), ECD showed a weak positive correlation with A2L (*ρ* = 0.316), suggesting that higher ECD may be associated with a more controlled recovery-phase deformation response during the second applanation.

Vertically across markers, ECD was significantly associated with nine biomechanical parameters (Figure 2B), dominated by deformation timing/length and central-dominant deformation (positive with A1V, A2L, DA Ratio; negative with IR and ARTh/cCBI), while HEX was significantly associated with 10 parameters (Figure 2C), dominated by stiffness-related response and controlled recovery (positive with SSI, SP-A1, A2L, A2V, and nctIOP) and by lower recovery-phase deformation response and lower structural risk (negative with DA, PD, IR, BAD. D, and cCBI).

#### A2L and cTBI among biomechanical predictors stably associated with endothelial abnormality

**Figure 3:**
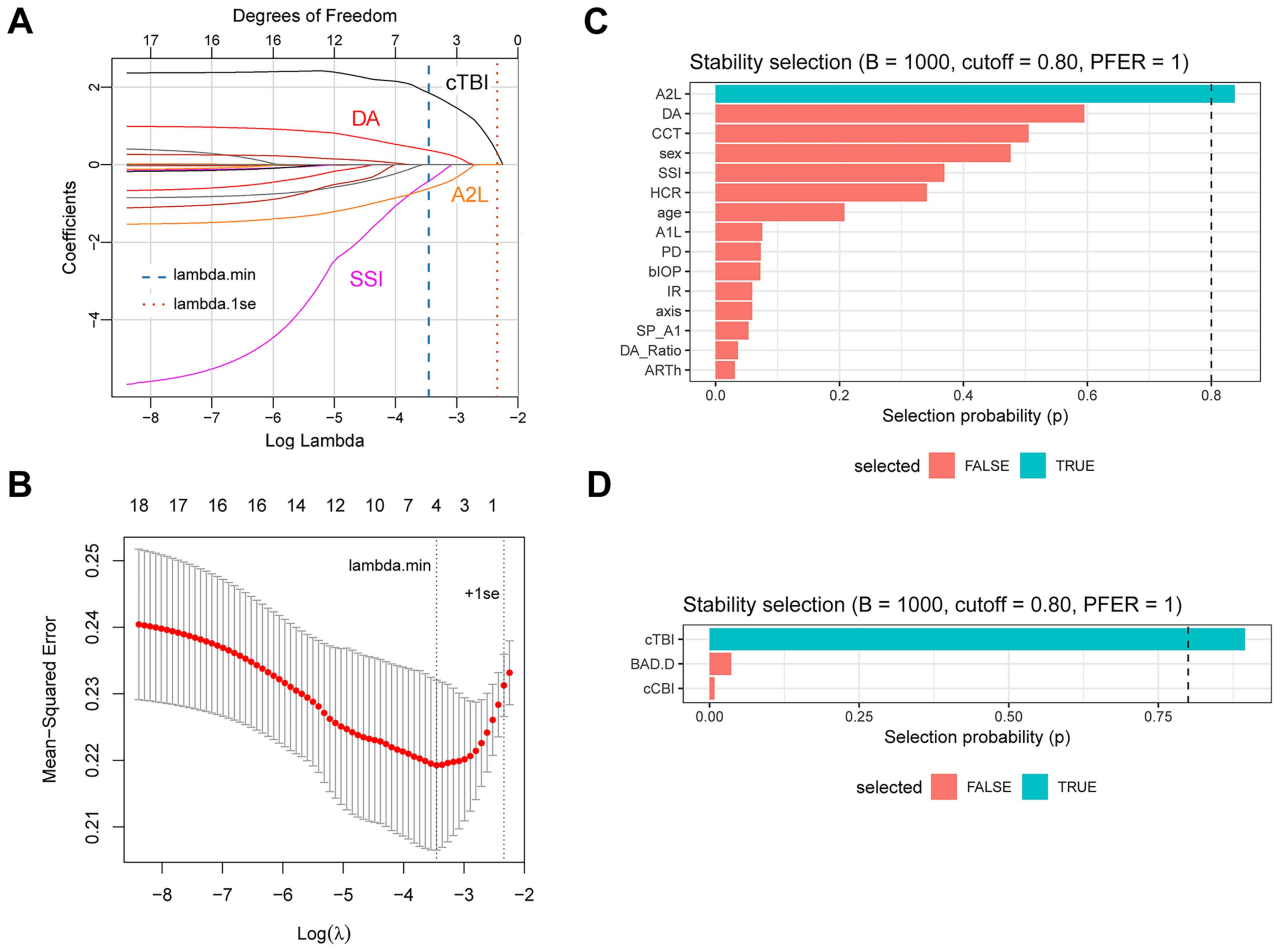
Regularized regression and stability selection of corneal biomechanical parameters. **(A)** LASSO coefficient paths. Logistic LASSO showing coefficient trajectories across the regularization path. The *x*-axis represents log(*λ*), and the top axis indicates effective degrees of freedom. The blue dashed line marks *λ*-at-min [minimum mean squared error (MSE)], and the orange dotted line marks *λ*.1se. Variables retained at *λ*-at-min are highlighted in color. **(B)** Cross-validation curve. Tenfold cross-validation curve plotting mean squared error (red dots) with ±1 standard error bars (gray). Vertical lines indicate *λ* at minimum MSE and *λ*-at-min + 1se. **(C)** Stability selection of raw features. Stability selection applied to raw biomechanical parameters (subsampling rate 0.5, *B* = 1,000, cutoff = 0.80, *PFER* ≤ 1). The horizontal axis shows selection probability (*π*); the dashed line represents the cutoff. A2L exceeded the cutoff and was stably selected. **(D)** Stability selection of composite indices. Stability selection applied separately to composite indices under the same settings. cTBI was the only parameter consistently selected. LASSO, Logistic regression with L1 penalty; *π*, proportion of times a variable is selected across subsamples; *PFER*, per-family error rate (upper bound on expected number of false selections).

Univariate analyses confirmed six parameters with between-group differences (Supplementary Table S2). To prioritize candidates, we applied LASSO regularization and ranked variables by their entry along the path (Figure 3A); four markers (only raw features rather than composite indices) were then taken forward to multivariable analysis. After adjustment for covariates, A2L remained the sole independent predictor of endothelial abnormality (*p* = 0.018) (Supplementary Table S2), consistent with the LASSO ranking.

To assess reproducibility beyond a single sample, we performed stability selection (subsampling rate 0.5; B = 1,000; cutoff = 0.80; PFER ≤ 1), analyzing raw features and composite indices in separate runs. This procedure identified A2L (raw set, see Figure 3C) and cTBI (composite set, see Figure 3D) as the only stable markers—thereby corroborating their diagnostic relevance under explicit error-control for false selections.

#### A targeted four-parameter biomechanical model enhances detection of endothelial compromise

In addition, a four-parameter model DASC (A2L, DA, SSI, CCT) was constructed based on variables selected from LASSO analysis and stability selection result. This composite model achieved a slightly higher AUC of 0.711 (95% CI: 0.614–0.808) compared to cTBI (Supplementary Figure S2, AUC: 0.683, 95%CI: 0.565–0.800), which was originally developed to detect keratoconus but showed a potential indicative value for abnormal endothelial status in this study (see Figure 3A, rank of variables along the regularization path), although the difference did not reach statistical significance (*p* > 0.05, DeLong’s test).

## Discussion

ECD decreases with age, yet most individuals (7) retain values above 2000 cells/mm^2^. Therefore, eyes with ECD below this range, although clinically clear, may represent a vulnerable subclinical state. Because invasive testing is inappropriate for transparent corneas, non-invasive biomechanical assessment may offer an opportunity to identify early endothelial compromise before decompensation becomes detectable.

A persistent limitation in previous work is the insufficient consideration of central corneal thickness as both a biomechanical modifier (30) and an endothelial correlate. CCT influences dynamic deformation behavior and may mask or distort genuine biomechanical–endothelial coupling (31–33) if not appropriately stratified. Few studies have attempted to identify independent predictors or construct reproducible diagnostic models for subclinical endothelial abnormalities.

Using an error-controlled machine-learning framework, we demonstrate that dynamic corneal response parameters can be distilled into a small, stable signature associated with idiopathic endothelial impairment. Stability selection allowed us to mitigate overfitting and control false discoveries, strengthening the reproducibility of A2L and cTBI as key discriminative parameters. The stromal matrix and endothelial layer function as a tightly coordinated unit (34); alterations in stromal hydration, collagen organization, or biomechanics can influence endothelial integrity and vice versa (35–37). Our findings extend this concept to a clinical population by showing that subtle, non-overt endothelial abnormalities are mirrored in macroscale biomechanical behavior.

Corneal biomechanical assessment utilizing ultra-high-speed Scheimpflug imaging coupled with machine learning algorithms enables non-invasive, multidimensional characterization of corneal biomechanics (21), this approach holds great significance to connect macroscale biomechanical profile with microstructural abnormalities, because if validated, these findings may provide clinically actionable insights with tangible applications in the management of corneal endothelial disorders.

The thinner corneas observed in the abnormal-endothelium group, though counterintuitive, align with literature (23, 38, 39) demonstrating a positive association between CCT and ECD, suggesting that low ECD may be accompanied by stromal thinning rather than reflecting a causal relationship. Another possible explanation is sampling bias due to limited numbers in the endothelial-abnormal group. Future studies with larger samples are needed to confirm whether corneal thinning is a surrogate marker of subclinical endothelial compromise or a coincidental finding in this high-risk population.

Because CCT exerts a confounding influence on biomechanics, propensity score matching was necessary to isolate its effect. After matching, several dynamic parameters remained significantly different. Lower SSI and HCR suggest reduced stromal stiffness-related response, while shorter A2L and A1T reflect diminished deformation response and accelerated initial deformation. Collectively, these features describe a cornea with reduced resistance, impaired structural coherence, and altered damping behavior—consistent with subclinical stromal changes secondary to endothelial dysfunction.

Correlation analysis further showed that biomechanical–endothelial coupling is strongly modulated by CCT. In mid-thickness corneas (530–570 μm), the relationships were most pronounced: eyes with better endothelial morphology exhibited greater stiffness-related response, more compact deformation, faster first applanation, and more controlled second-applanation recovery. These findings are consistent with the concept that early endothelial impairment may subtly alter stromal hydration and collagen architecture, producing detectable changes in deformation behavior before clinical edema develops (34, 40). The concentration of effects in Groups B and C suggests a physiological thickness range in which biomechanical signals most sensitively reflect endothelial status.

A2L consistently emerged as a protective factor across multiple analytic approaches. As a measure of the extent of corneal flattening during recoil, A2L reflects recovery-phase deformation response and stromal integrity. Reduced A2L has been reported in keratoconus (41, 42), post-crosslinking corneas (43), and high myopia (44), supporting its sensitivity to structural weakening. Our results suggest that endothelial dysfunction may likewise diminish deformation behavior, possibly through subtle stromal softening or disturbed fluid–collagen homeostasis.

The performance of cTBI, which was originally developed for keratoconus detection (45), may potentially serve as an exploratory indicator suggestive of abnormal endothelial status. Together with the DASC model, which incorporates CCT and selected dynamic response parameters, these findings support a possible link between corneal biomechanics and endothelial abnormality. However, as DASC did not significantly outperform cTBI, this interpretation remains exploratory and requires further validation.

In our study, although several eyes showed elevated cTBI values (Supplementary Figure S1), these eyes did not meet the clinical or tomographic diagnostic criteria for keratoconus or corneal ectasia on re-review. Therefore, values above ectasia-oriented thresholds should not be simply considered equivalent to a clinical diagnosis of ectasia, particularly in an elderly cataract cohort without clinical or tomographic evidence of keratoconus.

This study is limited by its cross-sectional design, relatively small abnormal-endothelium sample, lack of external validation for the predictive model, and reliance on morphological rather than functional endothelial assessment. In addition, the CCT-stratified analyses involved multiple comparisons which may increase the risk of type I error; therefore, these findings require validation in larger independent cohorts. The small number of abnormal-endothelium cases may increase the risk of sampling bias and statistical instability. Longitudinal and externally validated studies are required to determine whether the identified markers can predict future endothelial decompensation.

## Conclusion

Dynamic corneal biomechanics provide meaningful insight into idiopathic subclinical endothelial abnormalities. In this exploratory cohort, A2L and cTBI showed potential associations with abnormal endothelial status, and the DASC model provided an interpretable framework integrating CCT and selected dynamic response parameters. These findings demonstrate that biomechanical signatures may reflect early endothelial compromise, and highlight the potential of non-invasive biomechanical profiling for risk stratification during routine preoperative evaluation.

## Data Availability

The raw data supporting the conclusions of this article will be made available by the authors, without undue reservation.
