# Supplementary material for: Stability selection machine learning identifies biomechanical markers for subclinical idiopathic corneal endothelial abnormalities
Source: Front Med (Lausanne). 2026 Jul 28;13:1899851. doi: 10.3389/fmed.2026.1899851 (PMC13458755; doi:10.3389/fmed.2026.1899851)
Supplement: Supplementary file 1 [file Data_Sheet_1.DOCX]

**Figure S1 Comparative analysis of biomechanical profiles**. Boxplots illustrate six parameters from Corvis ST showing significant differences between patients with normal corneal endothelial cells (CEC) and those with abnormal CEC (n = 206 vs. 35). Eyes with endothelial abnormalities exhibited lower Stiffness Parameter at First Applanation (SSI), Highest Concavity Radius (HCR), Length of flattened cornea at second applanation (A2L) and Time of flattened cornea at first applanation (A1T), but higher corrected Tomographic Biomechanical Index (cTBI). P-values were calculated using appropriate group comparison tests and are shown above each plot.

**Figure S2. Receiver operating characteristic (ROC) curves of cTBI and DASC model** Receiver operating characteristic (ROC) curves comparing the performance of the proposed DASC model (orange line, Model 2) and the composite biomechanical index cTBI (blue line, Model 1) in identifying eyes with abnormal corneal endothelium. The area under the curve (AUC) was 0.711 (95% CI: 0.614–0.808) for DASC and 0.683 (95% CI: 0.565–0.800) for cTBI. Although not significantly different (p > 0.05, DeLong’s test), the DASC model showed a numerically higher AUC.
