# Supplementary material for: Stability selection machine learning identifies biomechanical markers for subclinical idiopathic corneal endothelial abnormalities
Source: Front Med (Lausanne). 2026 Jul 28;13:1899851. doi: 10.3389/fmed.2026.1899851 (PMC13458755; doi:10.3389/fmed.2026.1899851)
Supplement: Supplementary file 2 [file Table_1.DOCX]

**Supplementary Tables**

**Table S1. Continuous CCT interaction analysis for key biomechanical parameters**

| Parameter | Interaction | OR | 95% CI | P-value of interaction | Adjusted_covariates |
| --- | --- | --- | --- | --- | --- |
| A2L | A2L x CCT | 0.781 | 0.48-1.233 | 0.305 | age, bIOP |
| SSI | SSI x CCT | 0.951 | 0.618-1.489 | 0.822 | age, bIOP |
| HCR | HCR x CCT | 0.829 | 0.513-1.269 | 0.421 | age, bIOP |
| DA | DA x CCT | 1.004 | 0.829-1.346 | 0.489 | age, bIOP |
| A1T | A1T x CCT | 0.878 | 0.507-1.433 | 0.628 | age, bIOP |
| cTBI | cTBI x CCT | 1.219 | 0.879-1.721 | 0.243 | age, bIOP |

Logistic regression models were fitted separately for each biomechanical parameter. The outcome was abnormal corneal endothelial status. All continuous variables were standardized before model fitting. ORs represent the multiplicative interaction between each standardized biomechanical parameter and standardized CCT, adjusted for age and bIOP.

CCT, central corneal thickness

A2L, Length of flattened cornea at second applanation

SSI, Stiffness parameter at first applanation

HCR, Radius of curvature at highest concavity

DA, Deformation amplitude at apex

A1T, Time from start until first applanation

cTBI, corrected tomographic biomechanical index

**Table S2. Univariate and multivariate analysis of potential biomechanics markers of abnormal endothelial**

| Characteristics | Univariate | | Multivariate | | | |
| --- | --- | --- | --- | --- | --- | --- |
|  | OR | P | OR | 2.5% CI | 97.50% CI | P |
| cTBI | 15.64591 | 0.000013 |  |  |  |  |
| A2L | 0.206092 | 0.006775 | 0.201829 | 0.051185 | 0.730714 | 0.018053 |
| SSI | 0.106678 | 0.014716 | 0.399858 | 0.053263 | 2.821519 | 0.359333 |
| CCT | 0.986131 | 0.017368 | 0.990737 | 0.978047 | 1.003131 | 0.147503 |
| HCR | 0.515511 | 0.021017 |  |  |  |  |
| A1T | 0.802501 | 0.043757 |  |  |  |  |
| DA | 16.48891 | 0.062707 | 2.200391 | 0.842872 | 41.46808 | 0.350286 |

Logistic regression was performed to identify biomechanical parameters associated with endothelial abnormality. Four variables with statistical significance in univariate analysis or with clinical relevance were included in the multivariate model. Odds ratios (OR) and 95% confidence intervals (CI) are reported.

cTBI, corrected tomographic biomechanical index

A2L, Length of flattened cornea at second applanation

SSI, Stiffness parameter at first applanation

CCT, central corneal thickness

HCR, Radius of curvature at highest concavity

A1T, Time from start until first applanation

DA, Deformation amplitude at apex
